# Low-Carbohydrate, High-Protein, High-Fat Diets Rich in Livestock, Poultry and Their Products Predict Impending Risk of Type 2 Diabetes in Chinese Individuals that Exceed Their Calculated Caloric Requirement

**DOI:** 10.3390/nu10010077

**Published:** 2018-01-12

**Authors:** Ruiqi Shan, Wei Duan, Lei Liu, Jiayue Qi, Jian Gao, Yunlong Zhang, Shanshan Du, Tianshu Han, Xiuyu Pang, Changhao Sun, Xiaoyan Wu

**Affiliations:** National Key Discipline, Department of Nutrition and Food Hygiene, School of Public Health, Harbin Medical University, Harbin 150081, China; ruiqi0119@163.com (R.S.); mtybxs@163.com (W.D.); leier9211@163.com (L.L.); qijiayue678@163.com (J.Q.); gaojianerik@126.com (J.G.); 18946171537@163.com (Y.Z.); dushanshan1007@163.com (S.D.); snowcalendar@126.com (T.H.); pangxy123@163.com (X.P.)

**Keywords:** low-carbohydrate, high-fat and high-protein diets, type 2 diabetes, prospective study, extra energy intake, mediation analysis, insulin resistance

## Abstract

The evidence on the association between long-term low-carbohydrate, high-fat and high-protein diets and type 2 diabetes (T2D) is controversial. Until now, data is limited for Chinese populations, especially in considering the influence of extra energy intake. In this paper, we aimed to investigate the association of low-carbohydrate, high-fat and high-protein diets with type 2 diabetes (T2D) risk in populations consuming extra calories and those with normal caloric intake, We also determined whether the association is mediated by insulin resistance (IR) or β-cell dysfunction. A total of 3644 subjects in the Harbin People’s Health Study (Cohort 1, 2008–2012) and 7111 subjects in the Harbin Cohort Study on Diet, Nutrition and Chronic Non-Communicable Diseases (Cohort 2, 2010–2015) were analyzed, with a median follow-up of 4.2 and 5.3 years, respectively. Multivariate relative risks (RRs) and their 95% confidence intervals (95% CIs) were calculated to estimate the association between low-carbohydrate, high-fat and high-protein diet and T2D in logistic regression models. The multivariate RRs (95% CIs) were 1.00, 2.24 (1.07, 4.72) and 2.29 (1.07, 4.88) (*P*_trend_ = 0.04), and 1.00, 1.45 (0.91, 2.31) and 1.64 (1.03, 2.61) (*P*_trend_ = 0.04) across tertiles of low-carbohydrate, high-fat and high-protein diet scores in the population consuming extra calories in Cohort 1 and Cohort 2, respectively. The association was no longer significant after adjustment for livestock and its products, or poultry and its products. The mediation analysis discovered that this association in the population consuming extra calories was insulin resistance mediated, in both Cohort 1 and Cohort 2. However, the association was not significant among participants overall and participants with normal caloric intake. Our results indicated that long-term low-carbohydrate, high-fat and high-protein diets were associated with increased T2D risk among the population consuming extra calories, which may be caused by higher intake of animal-origin fat and protein as well as lower intake of vegetables, fruit and fiber. Additionally, the association was mediated by IR. In the population consuming extra calories, reducing the intake of livestock, poultry and their products and increasing the intake of vegetables, fruit and fiber might protect this population from developing T2D.

## 1. Introduction

The epidemic of type 2 diabetes (T2D) has been rapidly escalating worldwide [1], and this is attributed to a variety of behavioral and environmental risk factors [1]. Dietary factors are the major behavioral and environmental risk factors for T2D [1]. Previous short-term intervention studies show that low-carbohydrate diets played a protective role in diabetics’ weight loss and blood glucose stability [2,3,4]. However, evidence from epidemiological studies on the long-term effect of low-carbohydrate, high-fat and high-protein diets on the incidence of T2D is controversial [5,6,7]. As one macronutrient falls, another rises. Therefore, a low-carbohydrate, high-fat and high-protein diet score, considering the intake of carbohydrate, protein and fat simultaneously as the overall diet, has been used to represent long-term low carbohydrate, high-fat and high-protein dietary patterns, and this is calculated based on data from a food frequency questionnaire (FFQ) [5,6,7]. Low-carbohydrate, high-fat and high-protein diets have been associated with an increased risk of T2D incidence in Western populations [8], but are associated with a decreased risk in the incidence of T2D in Japanese populations [6]. Until now, data on the association of low-carbohydrate, high-fat and high-protein diets with the incidence of T2D is limited for the Chinese population, where the number of people with T2D is the highest in the world, accounting for 109.6 million in 2015 [9].

In addition, the Chinese are experiencing changing dietary patterns which is concomitant with excessive energy intake similar to that of westernized diets [10], even though the energy expenditure, as well as energy requirements have declined dramatically over the past few decades [11]. Appropriate energy intake is crucial in protecting oneself from chronic diseases [12,13]. However, the possible underlying mechanisms involved in the relationship between low carbohydrate, high-fat and high-protein diets and T2D risk, especially among populations consuming extra calories or populations with normal caloric intake, remain unclear.

The aims of the present study were to identify the relationship between low carbohydrate, high-fat and high-protein dietary patterns and the risk of T2D incidence in two Chinese cohort studies, estimate this relationship especially among populations consuming extra calories or populations with normal caloric intake, and evaluate the roles of foods and nutrients related to the association of low carbohydrate, high-fat and high-protein diets with T2D. Specifically, the strategy of mediation analysis was employed to quantify whether insulin resistance (IR) or β-cell dysfunction may contribute to the low carbohydrate, high-fat and high-protein diet-T2D relationship.

## 2. Materials and Methods

### 2.1. Study Population

The study populations were from the Harbin People’s Health Study (Cohort 1) and the Harbin Cohort Study on Diet, Nutrition and Chronic Non-Communicable Diseases (Cohort 2). The baseline survey of the Cohort 1 study consisting of 8940 people aged 20–74 years was finished in 2008. Information about demographic characteristics, dietary habits, and lifestyle was collected by trained healthcare workers using a structured questionnaire and a physical examination was conducted at the same time. Previous publications have described the baseline methods in more detail [14]. A total of 4515 individuals (50.5% of the total participants) were randomly selected for the follow-up survey due to limited financial resources, and 4158 participants completed the first follow-up survey with a response rate of 92.1%, in 2012. The baseline survey of Cohort 2 consisted of 9734 people aged 20–74 years and was completed in 2012 [15]. The baseline survey methods were the same as those in Cohort 1 and these have been previously described in detail. During 2015 to 2016, a total of 8913 participants finished the first follow-up survey with a response rate of 91.6%.

The present study consisted of 3644 participants from Cohort 1 and 7111 participants from Cohort 2 after excluding those who had T2D at the baseline survey and those whose energy intake values were extreme (men > 4200 or <800 kcal/day, women > 3500 or <500 kcal/day), and those whose body mass index (BMI), waist circumference were missing.

The Cohort 1 and Cohort 2 studies were reviewed by the institutional review boards of all institutes and were conducted in accordance with the Declaration of Helsinki. Written consents were obtained from all participants.

### 2.2. Dietary Assessment

A food frequency questionnaire (FFQ) was used at baseline to assess dietary intake over the past 12 months in the two studies, including 103 food items from 14 food groups (rice, wheat-containing foods, potato and its products, beans and their products, vegetables, fruits, livestock and its products, poultry and its products, dairy and its products, eggs and their products, fish and its products, snacks, beverages, and ice cream). The validity and reliability of the FFQ have been assessed in a previous study [14]. The Chinese Food Composition Tables were applied to calculate intakes of carbohydrate (in g/day), protein (in g/day), fat (in g/day), saturated fatty acid (in g/day), monounsaturated fatty acid (in g/day), cholesterol (in mg/day), and fiber (in g/day) [16]. Energy (in kcal/day) was calculated as:

Energy (kcal/day) = carbohydrate (g/day) × 4 + protein (g/day) × 4 + fat (g/day) × 9
(1)

### 2.3. Other Factors as Potential Confounders

Anthropometric indices were measured by trained medical staff according to a standard protocol, including weight (kg), height (m^2^), waist circumference (cm) and blood pressure (mmHg). BMI (kg/m^2^) was calculated as weight (kg) divided by the square of the height in meters (m^2^). Data on socio-demographic factors were collected, including age (years), sex (male/female), exercise regularity (any kind of recreational or sport physical activity other than walking for work or life performed three or more days per week for at least 30 min), level of education (no formal education, elementary school, middle/high school, technical school/college, postgraduate degree or above), family history of diabetes (yes/no), current smokers (smoked at least 100 cigarettes in their life time and smoke every day or some days now), current drinkers (consumed alcoholic drinks more than one time each month in the past 12 months), hypertension (systolic blood pressure ≥ 140 mmHg or diastolic blood pressure ≥ 90 mmHg, and/or taking medications for hypertension), and the presence of coronary heart disease at baseline was collected by using a structured questionnaire.

### 2.4. Biochemical Measurements and Outcome Ascertainment

An oral glucose tolerance test (OGTT) was carried out for each cohort participant according to the World Health Organization (WHO) guidelines [17]. Fasting and postprandial blood samples (fasting over 10 h and 2 h after drinking a 75 g glucose containing water) were collected for biochemical assessment. After collection, plasma samples were kept in a portable, insulated bag with ice packs (at about 0–4 °C) and were processed within 6 h for long-term storage at −80 °C. Fasting blood glucose and 2-h glucose was measured quantitatively with an auto-analyzer (Hitachi 7100 Auto-analyzer, Tokyo, Japan). Fasting insulin and 2-h insulin was measured by an immunofluorescence method (TOSOH automated enzyme immunoassay (EIA) analyzer AIA-2000ST, Tosoh Smd Inc., Grove, OH, USA). Homeostasis model assessment of insulin resistance (HOMA2-IR) and β-cell function (HOMA2-%B) were calculated by a HOMA2 Calculator, based on the plasma levels of fasting blood glucose and fasting insulin, which provide a more accurate representation of physiology and can successfully predict the homeostatic responses to an intravenous glucose infusion [18].

Based on the OGTT, the newly diagnosed T2D was defined as fasting blood glucose ≥ 7.0 mmol/L and/or 2-h glucose ≥ 11.1 mmol/L in both two studies.

### 2.5. Prediction of Energy Requirements

The population consuming extra calories was defined as those with energy intakes greater than their predicted energy requirements. Otherwise, participants were defined as the population with normal caloric intake. Predicted energy requirement was defined as basic metabolic rate (BMR) multiplied by physical activity level (PAL) [19]. The BMR was calculated [20] as:

for men, BMR (kcal/24 h) = 66.4730 + 13.7516 × weight (kg) + 5.0033 × height (cm) − 6.7550 × age (years)(2)

for women, BMR (kcal/24 h) = 655.0955 + 9.5634 × weight (kg) + 1.8496 × height (cm) − 4.6756 × age (years)(3)

The PAL was defined in terms of three levels of physical activity. Light, moderate, and high levels of physical activity were set at 1.55, 1.78, 2.1 for men and 1.56, 1.64, 1.82 for women, respectively [19].

### 2.6. Statistical Analysis

Selected baseline characteristics were compared between subjects with T2D and without T2D. *t*-tests were used for continuous variables and chi-square tests for categorical variables. To get the low-carbohydrate, high-fat and high-protein diet score, we divided the participants into 11 strata for each of the percentages of energy from carbohydrate, protein, and fat with equal sample sizes, respectively. For carbohydrate, participants in the highest stratum received a score of 0 and participants in the seventh stratum received 4 and so on down to the lowest stratum where participants received a score of 10. For protein and fat, the scoring was the same but the order was reversed. Participants in the lowest stratum received a score of 0 and participants in the seventh stratum received a 6 and so on down to the highest stratum where participants received a score of 10. The total scores were summed to create the low-carbohydrate, high-fat and high-protein diet score, which ranged from 0 (representing highest carbohydrate intakes, and lowest fat and protein intakes) to 30 (representing lowest carbohydrate intakes, and highest protein and fat intakes). Therefore, the higher the score, the more closely the participant followed a low-carbohydrate, high-fat and high-protein diet. We subdivided the score into three tertiles and we named them as High carbohydrate group (HiCHO), Moderate carbohydrate group (ModCHO) and Low carbohydrate group (LoCHO) from tertile 1 to tertile 3, respectively. In addition, we applied a carbohydrate:fat:protein % (CHO:FAT:PROT %) which was based on the average value of the energy supply ratio of three micronutrients across tetiles of low-carbohydrate, high-fat and high-protein diet scores in order to understand the terminology more intuitively.

Logistic regression models were applied to estimate the relative risk (RR) and their 95% confidence interval (95% CI) between tertile intakes of low-carbohydrate, high-fat and high-protein diets and T2D. Model 1 was adjusted for age at study recruitment (years) and sex (male/female). Model 2 was adjusted for age at study recruitment (years), sex (male/female), BMI (kg/m^2^), waist circumference (cm), current drinker (yes/no), current smoker (yes/no), education (7 categories), exercise regularly (yes/no), family history of diabetes (yes/no), hypertension (yes/no), coronary heart disease (yes/no), and total energy intake (kcal/day). To identify the effects of 14 foods groups and some nutrients (protein, fat, saturated fatty acid, monounsaturated fatty acid, and cholesterol) related to low-carbohydrate, high-fat and high-protein diets with T2D, we further adjusted for each of these in the multivariable adjustment models, respectively.

Mediation analysis was performed to evaluate the role of HOMA2-IR or HOMA2-%B as potential mediators of the association between low-carbohydrate, high-fat and high-protein diets and T2D. Statistical significance for the mediation effect was carried out by formally testing for the total effect and proportion via mediation [21,22].

Analyses were performed by using SPSS 21.0 (Beijing Stats Data Mining Co. Ltd., Beijing, China). Mediation analysis was carried out by adopting R version 3.0.3 (http://www.r-project.org/) and a two-sided *p*-value < 0.05 was considered statistically significant.

## 3. Results

### 3.1. Baseline Characteristics of Cohort 1 and Cohort 2

A total of 182 and 498 incident cases were identified during a median follow-up of 4.2 and 5.3 years for Cohort 1 and Cohort 2, respectively. The characteristics of participants at baseline in Cohort 1 and Cohort 2 are presented in Table 1. Compared with participants without T2D, participants with T2D were significantly older, had higher BMI, waist circumferences, fasting glucose, 2-h glucose, fasting insulin, 2-h insulin, HOMA2-IR, and had a higher prevalence of hypertension and coronary heart disease in both cohorts. In addition, participants with T2D tended to take less regular exercise in Cohort 1 and tended to be men in Cohort 2.

### 3.2. Nutrition Information across Tertiles of Low-Carbohydrate, High-Fat and High-Protein Diet Scorse

Nutrition information for participants at baseline across tertiles of low-carbohydrate, high-fat and high-protein diet scores in Cohort 1 and Cohort 2 are presented in Table 2. A low-carbohydrate, high-fat and high-protein diet was significantly associated with higher intakes of beans, livestock, poultry, fish, dairy, eggs, saturated fatty acids, monounsaturated fatty acids, and cholesterol, but lower intake of rice, wheat, potato, vegetable, fruit, fiber and energy intake in both studies. Also, the diet was associated with higher calculated energy requirements in Cohort 2.

### 3.3. Low-Carbohydrate, High-Protein and High-Fat Diet Score and the Risk of T2D Incidence in the Population Consuming Extra Calories or Population with Normal Caloric Intake

The low-carbohydrate, high-protein and high-fat diet score was associated with an increased risk of the incidence of T2D in the population consuming extra calories, after adjustment for potential confounding factors. Compared with participants in the lowest tertile, the RRs (95% CIs) for the second and third tertiles were 2.24 (1.07, 4.72) and 2.29 (1.07, 4.88) (*P*_trend_ = 0.04) in Cohort 1 and they were 1.45 (0.91, 2.31) and 1.64 (1.03, 2.61) (*P*_trend_ = 0.04) in Cohort 2, respectively. However, this association was not statistically significant in the overall population or in the population with normal caloric intake (Table 3 and Table 4).

### 3.4. The Food Groups Responsible for the Association between Low-Carbohydrate, High-Protein and High-Fat Diets and T2D among Populations Consuming Extra Calories

For further adjustment for food groups, the association of low-carbohydrate, high-fat and high-protein diets with T2D was no longer significant after adjustment for livestock and its products (*P*_trend_ = 0.06 in Cohort 1 and *P*_trend_ = 0.10 in Cohort 2) or poultry and its products (*P*_trend_ = 0.06 in Cohort 1 and *P*_trend_ = 0.12 in Cohort 2) in both cohorts. In addition, the association of low-carbohydrate, high-fat and high-protein diets with T2D was no longer significant after adjustment for egg and its products in Cohort 2 (*P*_trend_ = 0.13). After adjustment for other food groups, a low-carbohydrate, high-fat and high-protein diet was still significantly associated with increased risk of T2D incidence in both cohorts (Table 5). Moreover, livestock and its products, poultry and its products, and egg and its products were significant associated with nutrients such as protein, fat, saturated fatty acid, monounsaturated fatty acid and cholesterol (Table S1). The association between low-carbohydrate, high-fat and high-protein diets and T2D was not significant in either Cohort 1 or Cohort 2 after adjustment for protein, saturated fatty acid, and cholesterol, respectively. Similar results were found in the HCNNCDS after adjustment for fat and monounsaturated fatty acid, respectively (Table 5).

### 3.5. Mediation Analysis in the Relation between Tertiles of Low-Carbohydrate, High-Protein and High-Fat Diets and T2D

In the mediation assessment, statistically significant proportions via mediation effect of HOMA2-IR was observed in the relation between tertiles of low-carbohydrate, high-fat and high-protein diets and T2D in Cohort 1 (proportion via mediation and 95% CI is 0.072 (0.019, 0.225), *p* = 0.02) and Cohort 2 (proportion via mediation and 95% CI is 0.106 (0.038, 0.341), *p* < 0.01) (Table 6).

## 4. Discussion

In the present two population-based prospective cohort studies conducted in China, we found that low-carbohydrate, high-fat and high-protein diets were associated with increased risk of T2D incidence among the population consuming extra calories, which may be caused by higher intake of animal-origin fat and protein as well as lower intake of vegetable, fruit and fiber. Reducing the intake of livestock, poultry and their products and increasing the intake of vegetable and fruit might be a beneficial approach in this situation. In addition, we found that it was IR, and not β-cell dysfunction that mediated the effect of low-carbohydrate, high-fat and high-protein diets on T2D.

Low-carbohydrate, high-fat and high-protein diets were characterized with low intake of carbohydrate but high intakes of protein and fat. These diets may limit consumption of some healthy dietary components [23], such as whole grains, dietary fiber, phytochemicals, fruit and vegetables, which are associated with decreased risk of T2D [24,25,26], whereas they can be high in animal fat intake and red meat, which are associated with increased risk of T2D [27,28]. Positive associations between low-carbohydrate, high-fat and high-protein diets and T2D have been reported by De Koning et al. [8]. However, no statistically significant association between low-carbohydrate, high-fat and high-protein diets and T2D incidence was observed in the overall population, men, or women in the present study.

This difference might be influenced by dietary variation, such as in energy intake [8]. To elucidate whether energy intake influences the effect of low-carbohydrate, high-fat and high-protein diets on T2D, further analysis was conducted in the present study. It should be noted, energy intake itself cannot evaluate whether the participants take an appropriate amount of energy, which was defined as daily energy consumption being equal to energy requirements. Until now, no study has investigated the influence of inappropriate caloric intake on the association of low-carbohydrate, high-fat and high-protein diets with T2D incidence. In the present study, the association of low-carbohydrate, high-fat and high-protein diets with T2D incidence was evaluated in a population consuming extra calories and a population with normal caloric intake. We found that a low-carbohydrate, high-fat and high-protein diet was associated with increased risk of T2D incidence among the population consuming extra calories. However, this association was not observed among the population with normal caloric intake. Our data adds further support to the premise that prolonged extra caloric intake combined with low-carbohydrate, high-fat and high-protein diets may increase T2D risk.

In our study, higher intakes of livestock, poultry, and their products and lower intake of vegetable, fruit and fiber might be responsible for the association of low-carbohydrate, high-fat and high-protein diets with T2D among populations consuming extra calories. Animal sources of protein, fat (saturated fatty acid, monounsaturated fatty acid), and cholesterol are the main components of these foods which may increase T2D risk when their content is high. First, a higher dietary protein intake from animal origin is associated with the plasma level of branched-chain amino acids which may activate mTOR. This activation can lead to phosphorylation of the insulin receptor substrate 1, leading to decreased insulin sensitivity which provides a potential link between higher protein intake and insulin resistance or T2D risk [29]. Then, higher intake of total fat from animal origin, especially saturated fatty acids, may elevate plasma free fatty acid concentrations which inhibit glucose transport or phosphorylation and insulin stimulated peripheral glucose uptake, as well as decreasing muscle glycogen synthase activity. This induces insulin resistance [30] which might be related to an increased risk of T2D [31]. In addition, high circulating cholesterol levels increase cholesterol content in β-cell and reduced glucose-stimulated insulin secretion [32], which may increase the risk of T2D [33]. Furthermore, low-carbohydrate, high-protein, high-fat diets were associated with lower intake of whole grains, dietary fiber, fruit, vegetables in the present study. Lack of vegetables, fruit and fiber, which are rich in bioactive phytochemicals and antioxidants was also responsible for higher T2D risk among populations consuming extra calories. Fruit and vegetables have been shown to improve insulin sensitivity and insulin secretion to overcome insulin resistance in previous studies [24]. In particular, green leafy vegetables are rich in bioactive phytochemicals (such as vitamin C and carotenoids), which are known for their antioxidant properties and have been reported to protect against diabetes [34,35]. We also found that the HiCHO group of the population consuming extra calories was the group with the lowest incidence of diabetes and highest intake of fiber. It has been reported by previous studies that a high-carbohydrate high-fiber diet may improve tissue-insulin sensitivity and have a beneficial effect on glycemic control [36,37]. Fiber may contribute to decreasing T2D risk because of its low glycaemic load and high micronutrient content [24]. In addition, dietary fiber may improve the postprandial glycemic response and insulin concentrations by slowing the digestion and absorption of food and by regulating metabolic hormones [38,39]. Whether the association between low-carbohydrate, high-protein, high-fat diets and T2D was attributed to higher intake of livestock and protein or lower intake of vegetable, fruit and fiber needs random controlled clinical studies to confirm.

Given that IR and β-cell dysfunction play a major role in the pathophysiology of T2D [40,41,42,43], speculating that the association between low-carbohydrate, high-fat and high-protein diets and T2D should be partly mediated by IR and/or β-cell dysfunction is reasonable. The results of the mediation analysis indicated that the positive association of low-carbohydrate, high-fat and high-protein diets with T2D was mainly mediated by IR. The suggested mechanism for this is: in the high fat but low carbohydrate fed rat, fat oversupply increases the availability of intramuscular triglyceride, which may reduce the anti-lipolytic action of insulin, and the glucose-fatty acid cycle could be expected to lead to a reduction in glucose oxidation in muscle via production of acetyl CoA and inhibition of pyruvate dehydrogenase, which would result in insulin resistance [44,45].

The percentage of diabetes incidence was higher in Cohort 2 than that in Cohort 1 which may be due to the following reasons. Firstly, the average follow-up year of Cohort 2 was 5.3 years which is longer than 4.2 years for Cohort 1. So, there were more diabetes in Cohort 2. Secondly, the data from the National Bureau of Statistics of China shows that the Gross Domestic Product increased remarkably from 2008, which was 3.19 × 105 (in 100 million RMB) to 2012, which was 5.40 × 105 (in 100 million RMB) (http://data.stats.gov.cn/). The dramatic change in socio-economic conditions and infiltration of westernized diets may be associated with higher intake of livestock and poultry and lower intake of vegetables and fruit. In the present study of Cohort 1 and Cohort 2, the intake of livestock and poultry increased from 61 g/day to 67 g/day and 20 g/day to 25 g/day, respectively, and the intake of vegetables decreased from 286 g/day to 271 g/day (Table S2). That may be associated with a higher incidence of diabetes. In addition, it appears that participants in the high-fat group consumed less energy than the participants in the high-carbohydrate group which could be explained by the following reasons. Firstly, higher fat intake might have a higher satiating effect because of their slower digestion compared to higher carbohydrate intake [46]. Secondly, it might also be due to a prolonged suppression of intake under the higher fat diet compared with that under the higher carbohydrate diet [47]. Thirdly, the intake of carbohydrate is higher among the Chinese population and the energy supplied by carbohydrate is greater than fat and protein. Carbohydrate may play a major role in energy supply in our study.

There are several strengths in our study: the present two studies were population-based, prospectively designed with a high participation rate and follow-up rates; a wide range of potential confounders were adjusted in the present study; and the findings were validated in two independent studies. However, some limitations must be considered. First, although we adjusted for confounders, we cannot exclude the possibility of residual confounding. Second, BMR was calculated based on the Harris-Benedict equation in the present study, which may overestimate BMR. However, the Harris-Benedict equation can be conveniently calculated and may be suitable for large populations [48]. Third, the dietary data was collected only once and a more accurate depiction of dietary habits would be possible if it could be calibrated by a second dietary data survey. In addition, the participants in the two studies were all from north China and the findings of our study may not be directly generalizable to other populations.

## 5. Conclusions

We found that long-term low-carbohydrate, high-fat and high-protein diets increased the risk of T2D in the population consuming extra calories and this association was mediated by IR. Reducing the intake of livestock, poultry and their products and increasing the intake of vegetable, fruit and fiber might be a beneficial approach to this condition.

## Figures and Tables

**Table 1:** Selected baseline characteristics of the Harbin People’s Health Study (Cohort 1, 2008–2012) and the Harbin Cohort Study on Diet, Nutrition and Chronic Non-Communicable Diseases (Cohort 2, 2010–2015).

Variable	Cohort 1		Cohort 2	
	Diabetes (*n* = 182)	No Diabetes (*n* = 3462)	Diabetes (*n* = 498)	No Diabetes (*n* = 6613)
Age at recruitment (years)	54 ± 10	50 ± 10 *	53 ± 9	50 ± 9 *
Male (%)	34	32	43	33 *
BMI (kg/m^2^)	27 ± 4	25 ± 3 *	26 ± 3	25 ± 3 *
Waist circumference (cm)	88 ± 12	84 ± 10 *	89 ± 10	85 ± 10 *
Education (%)				
No formal education	3.7	1.9	2.6	1.3
Elementary school	9.8	5.4	4.7	5.0
Middle school	30.1	30.2	27.7	23.0
High school/secondary technical school	27.6	33.9	36.5	35.0
Technical school/college	28.2	27.9	28.1	34.9
Postgraduate degree or above	0.6	0.7	0.4	0.9
Exercised regularly (%)	56.2	67.1 *	46.7	46.4
Current smokers (%)	17.2	17.1	17.8	15.5
Current drinker (%)	34.4	37.9	33.8	34.8
Hypertension (%)	52.5	37.3 *	51.4	35.0 *
Coronary heart disease (%)	34.6	20.4 *	22.4	15.8 *
Family history of diabetes (%)	11.7	13.2	18.7	15.0
Actual energy intake (kcal/day)	2115 ± 655	2199 ± 653	2364 ± 755	2287 ± 660
Predicted energy requirement (kcal/day)	2243 ± 389	2230 ± 359	2305 ± 354	2227 ± 367
Fasting glucose (mmol/L)	5.1 ± 0.9	4.6 ± 0.6 *	5.2 ± 0.9	4.5 ± 0.7 *
2-h glucose (mmol/L)	6.8 ± 2.3	5.5 ± 1.5 *	7.1 ± 2.1	5.7 ± 1.6 *
Fasting insulin (µU/mL)	11.8 ± 10.4	8.0 ± 6.7 *	10.1 ± 6.5	8.1 ± 6.1 *
2-h insulin (µU/mL)	47.5 ± 40.4	35.7 ± 31.5 *	54.3 ± 42.0	42.3 ± 34.3 *
HOMA2-IR	1.0 ± 1.2	0.7 ± 0.8 *	0.8 ± 1.0	0.6 ± 0.8 *
HOMA2-%B	81.9 ± 89.2	82.1 ± 95.5	119.3 ± 63.2	124.3 ± 58.9

*: *p* < 0.05 when compared with non-cases. *t*-test were used for continuous variables; chi-square tests were used for categorical variables. Mean ± Standard Deviation were used for continuous variables. BMI: body mass index HOMA2-IR: Homeostasis model assessment of insulin resistance; HOMA2-%B: Homeostasis model assessment of β-cell function.

**Table 2:** Nutrition information across tertiles of low-carbohydrate, high-fat and high-protein diet scores in the Harbin People’s Health Study (Cohort 1, 2008–2012) and the Harbin Cohort Study on Diet Nutrition and Chronic Non-Communicable Diseases (Cohort 2, 2010–2015).

	HiCHO	ModCHO	LoCHO	*p* ^a^
**1. Cohort 1**				
** Ave CHO:FAT:PROT %**	**68:22:10**	**62:27:11**	**53:34:13**	
** Food Items**				
Rice (g/day)	269 ± 151	188 ± 103	133 ± 90	<0.05
Wheat (g/day)	138 ± 110	136 ± 105	118 ± 90	<0.05
Potato (g/day)	74 ± 78	51 ± 46	40 ± 39	<0.05
Bean (g/day)	36 ± 35	48 ± 54	56 ± 62	<0.05
Snack (g/day)	0.3 ± 0.5	0.2 ± 0.5	0.2 ± 0.4	0.13
Beverage (mL/day)	0.7 ± 1.9	0.6 ± 1.5	0.6 ± 1.2	0.35
Ice cream (g/day)	13 ± 37	12 ± 25	14 ± 45	0.55
Livestock (g/day)	32 ± 33	52 ± 44	94 ± 77	<0.05
Poultry (g/day)	11 ± 13	16 ± 20	32 ± 46	<0.05
Fish (g/day)	16 ± 21	24 ± 39	48 ± 105	<0.05
Dairy (g/day)	1.4 ± 1.8	1.9 ± 2.0	2.2 ± 2.3	<0.05
Egg (g/day)	33 ± 27	38 ± 28	46 ± 37	<0.05
Vegetable (g/day)	301 ± 215	300 ± 226	260 ± 215	<0.05
Fruit (g/day)	189 ± 186	147 ± 138	127 ± 107	<0.05
** Nutrient Items**				
Carbohydrate (g/day)	413 ± 122	330 ± 97	270 ± 97	<0.05
Fat (g/day)	58 ± 14	64 ± 16	77 ± 23	<0.05
Protein (g/day)	62 ± 19	61 ± 22	68 ± 32	<0.05
Saturated fatty acid (g/day)	11 ± 4	13 ± 4	18 ± 7	<0.05
Monounsaturated fatty acid (g/day)	16 ± 5	19 ± 6	25 ± 9	<0.05
Cholesterol (mg/day)	242 ± 166	294 ± 177	407 ± 248	<0.05
Fiber (g/day)	15 ± 7	13 ± 7	11 ± 6	<0.05
** Total Population**				
Participants consuming extra calories (%)	56.6	34.1	33.4	<0.05
Actual energy intake (kcal/day)	2419 ± 655	2136 ± 593	2048 ± 663	<0.05
Predicted energy requirement (kcal/day)	2209 ± 345	2239 ± 384	2242 ± 358	0.26
** Diabetes**				
Participants consuming extra calories (%)	38.8	43.1	31.7	0.43
Actual energy intake (kcal/day)	2307 ± 659	2186 ± 598	1907 ± 630	<0.05
Predicted energy requirement (kcal/day)	2318 ± 427	2242 ± 416	2186 ± 325	0.20
** No Diabetes**				
Participants consuming extra calories (%)	57.5	33.6	33.5	<0.05
Actual energy intake (kcal/day)	2424 ± 649	2133 ± 589	2056 ± 668	<0.05
Predicted energy requirement (kcal/day)	2198 ± 333	2239 ± 381	2250 ± 362	0.06
**2. Cohort 2**				
** Ave CHO:FAT:PROT %**	**69:21:10**	**61:27:11**	**52:34:14**	
** Food Items**				
Rice (g/day)	292 ± 153	201 ± 117	142 ± 97	<0.05
Wheat (g/day)	140 ± 115	135 ± 106	116 ± 89	<0.05
Potato (g/day)	80 ± 85	52 ± 50	44 ± 42	<0.05
Bean (g/day)	39 ± 43	47 ± 50	60 ± 69	<0.05
Snack (g/day)	0.3 ± 0.6	0.3 ± 0.6	0.2 ± 0.4	0.06
Beverage (mL/day)	0.7 ± 1.8	0.7 ± 1.7	0.7 ± 1.3	0.86
Ice cream (g/day)	15 ± 41	13 ± 30	17 ± 42	<0.05
Livestock (g/day)	36 ± 35	59 ± 51	101 ± 82	<0.05
Poultry (g/day)	13 ± 15	20 ± 24	39 ± 48	<0.05
Fish (g/day)	16 ± 20	24 ± 38	49 ± 99	<0.05
Dairy (g/day)	1.4 ± 1.8	1.9 ± 2.1	2.1 ± 2.3	<0.05
Egg (g/day)	32 ± 27	40 ± 31	54 ± 49	<0.05
Vegetable (g/day)	281 ± 208	287 ± 239	248 ± 209	<0.05
Fruit (g/day)	178 ± 179	150 ± 134	124 ± 107	<0.05
** Nutrient Items**				
Carbohydrate (g/day)	433 ± 118	342 ± 101	278 ± 100	<0.05
Fat (g/day)	60 ± 14	68 ± 17	82 ± 24	<0.05
Protein (g/day)	65 ± 19	64 ± 23	73 ± 32	<0.05
Saturated fatty acid (g/day)	122 ± 4	14 ± 5	19 ± 7	<0.05
Monounsaturated fatty acid (g/day)	17 ± 5	20 ± 6	26 ± 10	<0.05
Cholesterol (mg/day)	243 ± 167	316 ± 192	461 ± 318	<0.05
Fiber (g/day)	15 ± 6	14 ± 7	12 ± 6	<0.05
**Total Population**				
Participants consuming extra calories (%)	64.1	43.4	39.2	<0.05
Actual energy intake (kcal/day)	2528 ± 639	2232 ± 621	2139 ± 677	<0.05
Predicted energy requirement (kcal/day)	2199 ± 352	2235 ± 358	2259 ± 383	<0.05
** Diabetes**				
Participants consuming extra calories (%)	58.4	45.1	40.6	<0.05
Actual energy intake (kcal/day)	2585 ± 742	2287 ± 719	2257 ± 764	<0.05
Predicted energy requirement (kcal/day)	2237 ± 347	2284 ± 307	2373 ± 384	0.02
** No Diabetes**				
Participants consuming extra calories (%)	64.5	43.3	39.1	<0.05
Actual energy intake (kcal/day)	2527 ± 630	2227 ± 612	2128 ± 668	<0.05
Predicted energy requirement (kcal/day)	2197 ± 353	2231 ± 361	2249 ± 382	<0.05

^a^: ANOVA were used for continuous variables; chi-square tests were used for categorical variables. Mean ± Standard Deviation were used for continuous variables. HiCHO: High carbohydrate group; ModCHO: Moderate carbohydrate group; LoCHO: Low carbohydrate group; Ave: Average; CHO: energy supply ratio of carbohydrate; FAT: energy supply ratio of fat; PROT: energy supply ratio of protein.

**Table 3:** Relative risk (95% Confidence interval) of type 2 diabetes across tertiles of low-carbohydrate, high-fat and high-protein diet scores in the Harbin People’s Health Study (Cohort 1, 2008–2012).

	HiCHO	ModCHO	LoCHO	*P*_trend_
**1. Total Population (*n* = 3644)**				
** Ave CHO:FAT:PROT %**	**68:22:10**	**62:27:11**	**53:34:13**	
*n* (cases)	1172 (55) (4.7%)	1130 (57) (5.0%)	1342 (70) (5.2%)	
Model 1	1.00	1.14 (0.75, 1.73)	1.23 (0.82, 1.83)	0.32
Model 2	1.00	1.12 (0.72, 1.75)	1.30 (0.84, 2.01)	0.23
**2. Population Consuming Extra Calories (*n* = 1497)**				
** Ave CHO:FAT:PROT %**	**71:19:10**	**64:25:11**	**54:32:14**	
Nutrient (g/day)				
Carbohydrate	503 ± 106	434 ± 89	368 ± 81	<0.05
Fat	61 ± 12	74 ± 14	96 ± 21	<0.05
Protein	72 ± 16	78 ± 21	97 ± 33	<0.05
Fiber	18 ± 7	17 ± 8	15 ± 7	<0.05
Energy intake (kcal/day)				
All	2759 ± 532			
Actual	2850 ± 550	2714 ± 522	2724 ± 516	0.02
Predicted	2172 ± 298	2156 ± 331	2131 ± 308	0.41
BMI	24 ± 3	25 ± 3	25 ± 3	0.07
Fasting glucose (mmol/L)	4.6 ± 1.1	4.7 ± 1.3	4.7 ± 0.9	0.25
Fasting insulin (µU/mL)	8.0 ± 4.8	8.1 ± 4.1	8.3 ± 4.9	0.73
*n* (cases)	457 (13) (2.8%)	534 (29) (5.4%)	506 (26) (5.1%)	
Model 1	1.00	2.15 (1.04, 4.42)	1.98 (0.95, 4.13)	0.09
Model 2	1.00	2.24 (1.07, 4.72)	2.29 (1.07, 4.88)	0.04
**3. Population with Normal Caloric Intake (*n* = 2147)**				
** Ave CHO:FAT:PROT %**	**66:24:10**	**60:29:11**	**51:36:13**	
Nutrient (g/day)				
Carbohydrate	314 ± 66	275 ± 62	216 ± 63	<0.05
Fat	51 ± 9	59 ± 12	67 ± 16	<0.05
Protein	48 ± 12	51 ± 13	53 ± 17	<0.05
Fiber	11 ± 4	10 ± 5	9 ± 4	<0.05
Energy intake (kcal/day)				
All	1801 ± 402			
Actual	1903 ± 372	1834 ± 387	1682 ± 410	<0.05
Predicted	2290 ± 412	2284 ± 371	2281 ± 369	0.96
BMI	25 ± 4	25 ± 4	25 ± 3	0.26
Fasting glucose (mmol/L)	4.7 ± 1.1	4.7 ± 0.9	4.8 ± 1.2	0.26
Fasting insulin (µU/mL)	8.2 ± 4.7	8.0 ± 4.3	8.7 ± 5.0	0.05
*n* (cases)	701 (45) (6.4%)	656 (28) (4.3%)	790 (41) (5.2%)	
Model 1	1.00	0.62 (0.36, 1.06)	0.81 (0.50, 1.31)	0.33
Model 2	1.00	0.68 (0.39, 1.20)	0.81 (0.47, 1.39)	0.44

Model 1 adjusted for age, sex. Model 2 adjusted for age, sex, body mass index, waist circumference, current drinkers, current smokers, education, exercise regularly, family history of diabetes, coronary heart disease, hypertension, and total energy intake.

**Table 4:** Relative risk (95% Confidence interval) of type 2 diabetes across tertiles of low-carbohydrate, high-fat and high-protein diet scores in the Harbin Cohort Study on Diet Nutrition and Chronic Non-Communicable Diseases (Cohort 2, 2010–2015).

	HiCHO	ModCHO	LoCHO	*P*_trend_
**1. Total Population (*n* = 7111)**				
** Ave CHO:FAT:PROT %**	**69:21:10**	**61:27:12**	**52:34:14**	
*n* (cases)	2265 (148) (6.5%)	2266 (155) (6.8%)	2580 (195) (7.6%)	
Model 1	1.00	1.09 (0.80, 1.49)	1.26 (0.94, 1.70)	0.12
Model 2	1.00	1.18 (0.85, 1.63)	1.30 (0.94, 1.79)	0.11
**2. Population Consuming Extra Calories** **(*n* = 3448)**				
** Ave CHO:FAT:PROT %**	**70:20:10**	**63:25:12**	**53:33:14**	
Nutrient (g/day)				
Carbohydrate	503 ± 96	429 ± 85	364 ± 82	<0.05
Fat	64 ± 13	78 ± 15	98 ± 22	<0.05
Protein	72 ± 17	80 ± 20	97 ± 30	<0.05
Fiber	18 ± 6	17 ± 7	15 ± 7	<0.05
Energy intake (kcal/day)				
All	2779 ± 521			
Actual	2870 ± 518	2731 ± 512	2733 ± 523	<0.05
Predict	2163 ± 320	2166 ± 316	2160 ± 311	0.96
BMI	25 ± 4	25 ± 4	24 ± 3	0.07
Fasting glucose (mmol/L)	4.6 ± 1.0	4.7 ± 1.2	4.7 ± 1.1	0.23
Fasting insulin (µU/mL)	7.9 ± 4.2	8.3 ± 4.5	8.3 ± 4.7	0.32
*n* (cases)	1167 (71) (6.1%)	1115 (77) (6.9%)	1166 (88) (7.6%)	
Model 1	1.00	1.30 (0.83, 2.03)	1.45 (0.94, 2.24)	0.10
Model 2	1.00	1.45 (0.91, 2.31)	1.64 (1.03, 2.61)	0.04
**3. Population with Normal Caloric Intake (*n* = 3663)**				
** Ave CHO:FAT:PROT %**	**66:24:10**	**59:30:11**	**50:37:13**	
Nutrient (g/day)				
Carbohydrate	321 ± 69	272 ± 63	219 ± 68	<0.05
Fat	51 ± 10	60 ± 12	71 ± 18	<0.05
Protein	49 ± 12	51 ± 15	56 ± 20	<0.05
Fiber	12 ± 4	11 ± 5	9 ± 5	<0.05
Energy intake (kcal/day)				
All	1835 ± 423			
Actual	1945 ± 384	1832 ± 394	1740 ± 456	<0.05
Predict	2277 ± 397	2284 ± 374	2328 ± 416	0.04
BMI	26 ± 4	26 ± 3	26 ± 4	0.88
Fasting glucose (mmol/L)	4.6 ± 1.1	4.6 ± 1.1	4.6 ± 1.1	0.92
Fasting insulin (µU/mL)	8.3 ± 4.7	8.5 ± 5.0	8.8 ± 5.1	0.37
*n* (cases)	1163 (80) (6.9%)	1184 (84) (7.1%)	1316 (98) (7.4%)	
Model 1	1.00	1.06 (0.69, 1.61)	1.16 (0.77, 1.74)	0.48
Model 2	1.00	1.01 (0.65, 1.57)	1.11 (0.71, 1.72)	0.64

Model 1 adjusted for age, sex. Model 2 adjusted for age, sex, body mass index, waist circumference, current drinkers, current smokers, education, exercise regularly, family history of diabetes, coronary heart disease, hypertension, and total energy intake.

**Table 5:** Relative risk (95% Confidence interval) of type 2 diabetes across tertiles of low-carbohydrate, high-fat and high-protein diet scores after adjustment for foods and nutrients in the population consuming extra calories of the Harbin People’s Health Study (Cohort 1, 2008–2012) and Harbin Cohort Study on Diet Nutrition and Chronic Non-Communicable Diseases (Cohort 2, 2010–2015).

	HiCHO	ModCHO	LoCHO	*P*_trend_
**1. Cohort 1**				
Low-carbohydrate, high-fat and high-protein diet score	1.00	2.24 (1.07, 4.72)	2.29 (1.07, 4.87)	<0.05
** Food Items**				
Adjusted for rice	1.00	2.28 (1.08, 4.81)	2.58 (1.17, 5.72)	<0.05
Adjusted for wheat	1.00	2.22 (1.05, 4.67)	2.29 (1.07, 4.89)	<0.05
Adjusted for potato	1.00	2.24 (1.06, 4.74)	2.32 (1.07, 5.07)	<0.05
Adjusted for bean	1.00	2.18 (1.02, 4.66)	2.26 (1.04, 4.88)	<0.05
Adjusted for snack	1.00	2.40 (1.13, 5.10)	2.46 (1.14, 5.29)	<0.05
Adjusted for beverage	1.00	2.24 (1.07, 4.73)	2.34 (1.09, 5.02)	<0.05
Adjusted for ice cream	1.00	2.31 (1.09, 4.88)	2.38 (1.11, 5.12)	<0.05
Adjusted for livestock	1.00	2.12 (1.02, 4.40)	2.27 (0.96, 5.30)	0.06
Adjusted for poultry	1.00	2.22 (1.05, 4.66)	2.21 (0.99, 4.90)	0.06
Adjusted for fish	1.00	2.15 (1.02, 4.52)	2.25 (1.03, 4.90)	<0.05
Adjusted for dairy	1.00	2.24 (1.04, 4.85)	2.33 (1.06, 5.14)	<0.05
Adjusted for egg	1.00	2.19 (1.04, 4.62)	2.24 (1.04, 4.82)	<0.05
Adjusted for vegetable	1.00	2.23 (1.06, 4.70)	2.31 (1.07, 4.90)	<0.05
Adjusted for fruit	1.00	2.20 (1.05, 4.64)	2.28 (1.07, 4.88)	<0.05
** Nutrient Items**				
Adjusted for protein	1.00	2.11 (1.00, 4.44)	1.80 (0.78, 4.17)	0.18
Adjusted for fat	1.00	2.42 (1.13, 5.50)	2.75 (1.18, 6.46)	<0.05
Adjusted for saturated fatty acid	1.00	2.35 (1.04, 5.30)	2.54 (0.86, 7.51)	0.09
Adjusted for monounsaturated fatty acid	1.00	2.43 (1.16, 5.63)	2.83 (1.26, 6.33)	<0.05
Adjusted for cholesterol	1.00	2.07 (0.98, 4.40)	1.80 (0.78, 4.20)	0.18
Adjusted for fiber	1.00	2.10 (1.00, 4.45)	2.25 (1.05, 4.83)	<0.05
**2. Cohort 2**				
Low-carbohydrate, high-fat and high-protein diet score	1.00	1.45 (0.91, 2.31)	1.64 (1.03, 2.61)	<0.05
** Food Items**				
Adjusted for rice	1.00	1.46 (0.92, 2.33)	1.67 (1.03, 2.74)	<0.05
Adjusted for wheat	1.00	1.45 (0.91, 2.31)	1.64 (1.03, 2.60)	<0.05
Adjusted for potato	1.00	1.44 (0.98, 2.29)	1.63 (1.03, 2.59)	<0.05
Adjusted for bean	1.00	1.44 (0.90, 2.31)	1.63 (1.01, 2.61)	<0.05
Adjusted for snack	1.00	1.48 (0.93, 2.36)	1.65 (1.04, 2.63)	<0.05
Adjusted for beverage	1.00	1.45 (0.91, 2.30)	1.64 (1.03, 2.60)	<0.05
Adjusted for ice cream	1.00	1.45 (0.91, 2.31)	1.64 (1.03, 2.60)	<0.05
Adjusted for livestock	1.00	1.43 (0.88, 2.32)	1.59 (0.91, 2.81)	0.10
Adjusted for poultry	1.00	1.39 (0.87, 2.24)	1.49 (0.89, 2.49)	0.12
Adjusted for fish	1.00	1.45 (0.91, 2.31)	1.64 (1.01, 2.65)	<0.05
Adjusted for dairy	1.00	1.52 (0.95, 2.43)	1.75 (1.09, 2.80)	<0.05
Adjusted for egg	1.00	1.37 (0.85, 2.19)	1.49 (0.93, 2.40)	0.13
Adjusted for vegetable	1.00	1.42 (0.89, 2.26)	1.63 (1.03, 2.60)	<0.05
Adjusted for fruit	1.00	1.48 (0.93, 2.35)	1.70 (1.10, 2.70)	<0.05
** Nutrient Items**				
Adjusted for protein	1.00	1.34 (0.83, 2.17)	1.35 (0.76, 2.40)	0.30
Adjusted for fat	1.00	1.20 (0.71, 2.03)	1.05 (0.50, 2.23)	0.84
Adjusted for saturated fatty acid	1.00	1.29 (0.77, 2.18)	1.25 (0.60, 2.62)	0.51
Adjusted for monounsaturated fatty acid	1.00	1.26 (0.76, 2.11)	1.18 (0.58, 2.39)	0.62
Adjusted for cholesterol	1.00	1.36 (0.85, 2.17)	1.36 (0.81, 2.30)	0.24
Adjusted for fiber	1.00	1.44 (0.91, 2.29)	1.71 (1.07, 2.72)	<0.05

Models were adjusted for age, sex, body mass index, waist circumference, current drinkers, current smokers, education, exercise regularly, family history of diabetes, coronary heart disease, hypertension, total energy intake, indicated foods, and indicated nutrients.

**Table 6:** The effect (95% Confidence interval) of tertiles of low-carbohydrate, high-fat and high-protein diet scores on type 2 diabetes with mediation of biomarkers in the population consuming extra calories of the Harbin People’s Health Study (Cohort 1, 2008–2012) and Harbin Cohort Study on Diet Nutrition and Chronic Non-Communicable Diseases (Cohort 2, 2010–2015).

Mediators	Total Effect Estimate	Proportion via Mediation Estimate	Sensitivity Analysis	
			*R*^2^ *	${\tilde{R}}^{2}$
**1. Cohort 1**				
HOMA2-IR	0.017 (0.008, 0.026)	0.072 (0.019, 0.225)	0.01	0.008
HOMA2-%B	0.019 (0.008, 0.026)	−0.005 (−0.167, 0.047)	NA	NA
**2. Cohort 2**				
HOMA2-IR	0.015 (0.007, 0.021)	0.106 (0.038, 0.341)	0.01	0.008
HOMA2-%B	0.013 (0.002, 0.020)	0.131 (−0.280, 0.456)	NA	NA

Models were adjusted for age, sex, body mass index, waist circumference, current drinkers, current smokers, education, exercise regularly, family history of diabetes, coronary heart disease, hypertension, and total energy intake. *R*^2^ *: the proportion of residual variances that were explained by the omitted confounding. ${\tilde{R}}^{2}$: the proportion of total variances that were explained by the omitted confounding; NA: not available.

## Supplementary Materials

The following are available online at http://www.mdpi.com/2072-6643/10/1/77/s1, Table S1, Pearson’s correlations between livestock, poultry and their products and relevant nutrients in the Harbin People’s Health Study (Cohort 1, 2008–2012) and Harbin Cohort Study on Diet, Nutrition and Chronic Non-Communicable Diseases (Cohort 2, 2010–2015); Table S2, Selected baseline food intake of the Harbin People’s Health Study (Cohort 1, 2008–2012) and Harbin Cohort Study on Diet, Nutrition and Chronic Non-Communicable Diseases (Cohort 2, 2010–2015).
