# Supplementary material for: Low-Carbohydrate, High-Protein, High-Fat Diets Rich in Livestock, Poultry and Their Products Predict Impending Risk of Type 2 Diabetes in Chinese Individuals that Exceed Their Calculated Caloric Requirement
Source: Nutrients. 2018 Jan 12;10(1):77. doi: 10.3390/nu10010077 (PMC5793305; doi:10.3390/nu10010077)
Supplement: Supplementary file 1 [file nutrients-10-00077-s001.pdf]

Table S1 Pearson's correlations between livestock, poultry, egg and their products and relevant nutrients in the Harbin People's Health Study (Cohort 1, 2008-2012) and Harbin Cohort Study on Diet, Nutrition and Chronic Non-communicable Diseases (Cohort 2, 2010-2015)

Nutrients	Foods		
	Livestock	Poultry	Egg
**Cohort 1**			
Protein	0.320**	0.381**	0.326**
Fat	0.786**	0.437**	0.288**
Saturated fatty acid	0.849**	0.474**	0.342**
Monounsaturated fatty acid	0.853**	0.521**	0.255**
Cholesterol	0.261**	0.362**	0.919**
**Cohort 1**			
Protein	0.407**	0.424**	0.352**
Fat	0.784**	0.508**	0.309**
Saturated fatty acid	0.849**	0.535**	0.366**
Monounsaturated fatty acid	0.857**	0.565**	0.276**
Cholesterol	0.266**	0.346**	0.942**

** All displayed correlations were significant at P <0.01.

Table S2 Selected baseline food intake of the Harbin People's Health Study (Cohort 1, 2008-2012) and Harbin Cohort Study on Diet, Nutrition and Chronic Non-communicable Diseases (Cohort 2, 2010-2015)

Food items(g/day)	Cohort 1	Cohort 2
Livestock	61±62	67±66
Poultry	20±32	25±34
Vegetable	286±219	271±219

Mean ± Standard Deviation were used for continuous variables.
